# Supplementary material for: Rescuing Perishable Neuroanatomical Information from a Threatened Biodiversity Hotspot: Remote Field Methods for Brain Tissue Preservation Validated by Cytoarchitectonic Analysis, Immunohistochemistry, and X-Ray Microcomputed Tomography
Source: PLoS One. 2016 May 19;11(5):e0155824. doi: 10.1371/journal.pone.0155824 (PMC4873048; doi:10.1371/journal.pone.0155824)
Supplement: S2 File — (DOCX) [file pone.0155824.s002.docx]

setwd("C:\\UTEP\\SCL\\Danny\\")

# BRING IN THE DATA

dat <- read.csv(file="dat-glmm.csv", header=T)

head(dat); colnames(dat)

dim(dat)

# ================================

# GEE ANALYSIS OF ORIGINAL DATA

# =================================

# install.packages("geepack")

library(geepack)

# help(package="geepack")

dat <- dat[order(dat$Slide), ]

y <- dat$Y1

y <- factor(y, ordered=T)

fit <- ordgee(y ~ Method + factor(Observer),

id=Slide, data=dat,

mean.link = "logit", corstr = "independence",

control= geese.control(epsilon=1e-06, maxit=100))

summary(fit)

# =======================================

# GEE ANALYSIS OF THE AGGREGATED DATA

# =======================================

Mode <- function(x) {

ux <- unique(x)

ux[which.max(tabulate(match(x, ux)))]

}

defactor <- function(x) as.numeric(levels(x))[x]

dat0 <- aggregate(Y1~Observer + Slide, data=dat, FUN=Mode)

dat0 <- as.data.frame(dat0)

dat0

dat0$Slide <- as.character(dat0$Slide)

table(dat$Method, dat$Slide)

table(dat$Animal, dat$Slide)

method1 <- scan()

30 31 32 33 34 35 36 37 38 39

dat0$Method <- ifelse(is.element(dat0$Slide, method1), 1, 0)

dat0 <- dat0[order(dat0$Slide), ]

# dat0 <- dat0[order(dat0$Slide), ]

# write.csv(dat0, file="dat0.csv")

dat0$Animal <- "C"

dat0$Animal[is.element(dat0$Slide, 1:6)] <- "A"

dat0$Animal[is.element(dat0$Slide, 7:9)] <- "B"

dat0$Animal[is.element(dat0$Slide, 24:29)] <- "D"

dat0$Animal[is.element(dat0$Slide, 30:37)] <- "S"

dat0$Animal[dat0$Slide=="38"] <- "T"

dat0$Animal[dat0$Slide=="39"] <- "U"

dat0

# VARY THROUGH Y2-Y6

dat.tmp <- aggregate(Y6~Observer + Slide, data=dat, FUN=Mode)

dat.tmp$Slide <- as.character(dat.tmp$Slide)

dat.tmp <- dat.tmp[order(dat.tmp$Slide), ]

dat0$Y6 <- dat.tmp$Y6

# write.csv(dat0, file="dat0.csv")

# GEE ANALYSIS

dat <- read.csv(file="dat0.csv", header=T)

library(geepack)

# help(package="geepack")

y <- dat$Y5

y <- factor(y, ordered=T)

fit <- ordgee(y ~ Method + factor(Observer),

id=Animal, data=dat,

mean.link = "logit", corstr = "independence",

control= geese.control(epsilon=1e-04, maxit=25))

summary(fit)

dat <- dat[order(dat$Slide), ]

vnames <- colnames(dat); vnames

y.cols <- 5:10

OUT <- NULL

for(j in 1:length(y.cols)){

y <- dat[, y.cols[j]]

yname <- vnames[y.cols[j]]

y <- factor(y, ordered=T)

fit <- ordgee(y ~ Method + factor(Observer),

id=Slide, data=dat,

mean.link = "logit", corstr = "independence",

control= geese.control(epsilon=1e-04, maxit=25))

beta.Method <- fit$beta[3]

se.Method <- sqrt(diag(fit$vbeta))[3]

z.Method <- beta.Method/se.Method

p.value <- 2*pnorm(abs(z.Method), lower.tail =F)

out <- c(var=yname, beta.Method=beta.Method, se.Method=se.Method, z.Method=z.Method, p.value=p.value)

OUT <- rbind(OUT, out)

}

row.names(OUT) <- NULL

OUT <- as.data.frame(OUT)

OUT

write.csv(OUT, file="results.csv", row.names=F)

# HEATMAP

# =============

dat.heat <- read.csv(file="dat-heatmap.csv", header=F)

head(dat.heat)

colnames(dat.heat) <- c("", "Y1", "", "", "Y2", "", "", "Y3", "",

"", "Y4", "", "", "Y5", "", "", "Y6", "")

# ?heatmap

heatmap(x=as.matrix(dat.heat), Colv = NA, Rowv=NA,

col = gray.colors(16), scale = "none", revC=T,

margins = c(5, 5), labRow=NULL, labCol =NULL,

xlab = "", ylab = "",

main = "Heatmap")

topo.colors(16)

cm.colors(256)

#
